# [2-(4-Methyl­piperazin-1-ylmeth­yl)phen­yl]diphenyl­phosphane

**DOI:** 10.1107/S1600536809049526

**Published:** 2009-11-25

**Authors:** Ancuţa Covaci, Ciprian I. Raţ, Cristian Silvestru

**Affiliations:** a Universitatea Babeş-Bolyai, Facultatea de Chimie şi Inginerie Chimicã, 11 Arany Janos, 400028 Cluj-Napoca, Romania

## Abstract

In the title compound, C_24_H_27_N_2_P, the P atom is bonded to three C atoms in a trigonal–pyramidal geometry. The overall Ψ-trigonal-bipyramidal coordination of the P atom is established when the contribution of the electron lone pair and of the N—P donor–acceptor distance of 3.051 (3)Å are considered. The 4-methyl­piperazinyl ring adopts a chair conformation. Intra- and inter­molecular C—H⋯π hydrogen bonding leads to the consolidation of the structure.

## Related literature

For organophospho­rus compounds containing substituents with the capability of intra­molecular donor⋯acceptor inter­actions, see: Alberico *et al.* (2007 ▶); Chandrasekaran *et al.* (2002 ▶); Chuit *et al.* (1993 ▶); Pretorius *et al.* (2004 ▶). For the structures of triclinic polymorphs of triphenyl­phosphine, see: Ziemer *et al.* (2000 ▶).
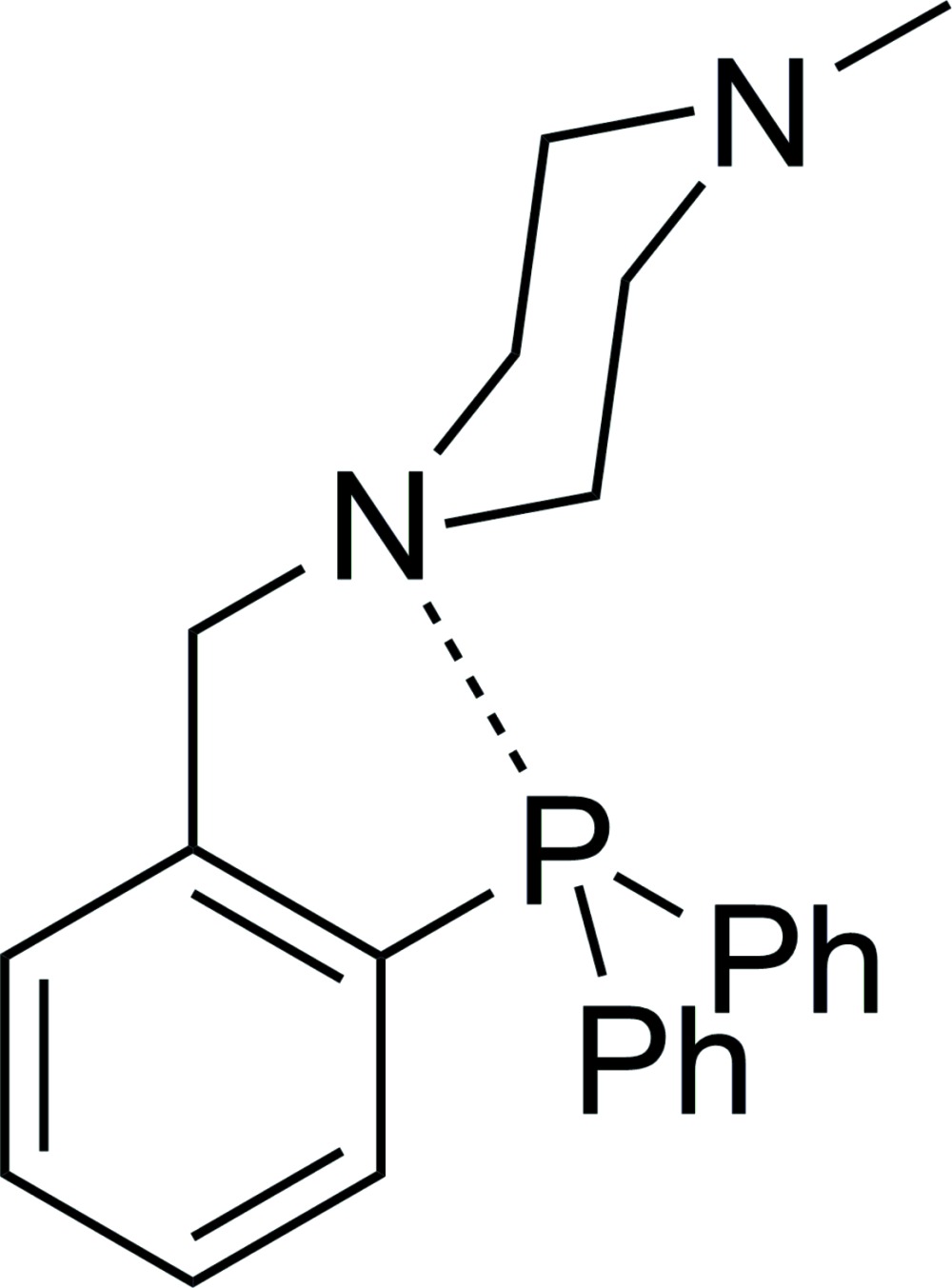


## Experimental

### 

#### Crystal data

C_24_H_27_N_2_P
*M* *_r_* = 374.45
Monoclinic, 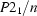

*a* = 9.3689 (10) Å
*b* = 14.6735 (16) Å
*c* = 15.4362 (16) Å
β = 100.849 (2)°
*V* = 2084.2 (4) Å^3^
*Z* = 4
Mo *K*α radiation
μ = 0.14 mm^−1^
*T* = 297 K
0.30 × 0.26 × 0.21 mm

#### Data collection

Bruker SMART APEX CCD area-detector diffractometer
Absorption correction: multi-scan (*SADABS*; Bruker, 2000 ▶) *T*_min_ = 0.959, *T*_max_ = 0.971
14906 measured reflections
3667 independent reflections
2811 reflections with *I* > 2σ(*I*)
*R*_int_ = 0.064

#### Refinement

*R*[*F* ^2^ > 2σ(*F* ^2^)] = 0.085
*wR*(*F* ^2^) = 0.178
*S* = 1.20
3667 reflections
245 parameters
H-atom parameters constrained
Δρ_max_ = 0.25 e Å^−3^
Δρ_min_ = −0.25 e Å^−3^

### 

Data collection: *SMART* (Bruker, 2000 ▶); cell refinement: *SAINT-Plus* (Bruker, 2001 ▶); data reduction: *SAINT-Plus*; program(s) used to solve structure: *SHELXS97* (Sheldrick, 2008 ▶); program(s) used to refine structure: *SHELXL97* (Sheldrick, 2008 ▶); molecular graphics: *DIAMOND* (Brandenburg, 2009 ▶); software used to prepare material for publication: *publCIF* (Westrip, 2009 ▶).

## Supplementary Material

Crystal structure: contains datablocks I, global. DOI: 10.1107/S1600536809049526/wm2285sup1.cif

Structure factors: contains datablocks I. DOI: 10.1107/S1600536809049526/wm2285Isup2.hkl

Additional supplementary materials:  crystallographic information; 3D view; checkCIF report

## Figures and Tables

**Table:** 

C1—P1	1.841 (4)
C13—P1	1.829 (4)
C19—P1	1.839 (4)

**Table:** 

C13—P1—C19	101.62 (16)
C13—P1—C1	103.38 (16)
C19—P1—C1	100.26 (16)

**Table 2:** Hydrogen-bond geometry (Å, °)

*D*—H⋯*A*	*D*—H	H⋯*A*	*D*⋯*A*	*D*—H⋯*A*
C9—H9*B*⋯*Cg*3	0.97	2.91	3.835 (4)	160
C14—H14⋯*Cg*4^i^	0.97	2.74	3.651 (5)	166
C23—H23⋯*Cg*2^ii^	0.97	2.98	3.830 (5)	154

Symmetry codes: (i) 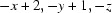; (ii) 
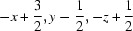
. *Cg*2, *Cg*3, and *Cg*4 are the centroids of the C1–C6, C13–C18, and C19–C24 benzene rings, respectively.

**Fig. 1.**
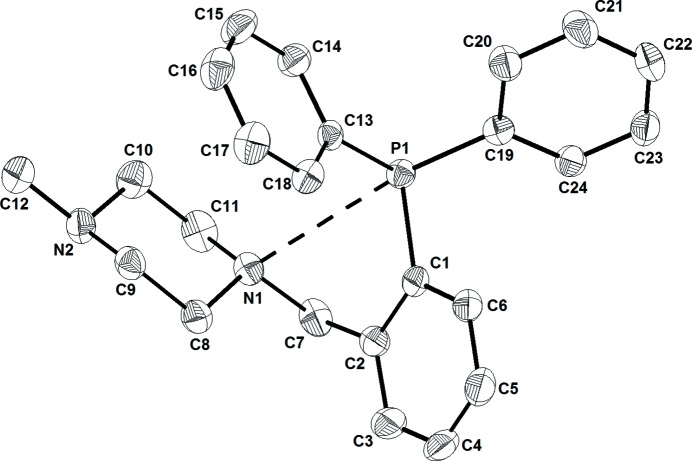
Graphical representation of the molecular structure of RNAP-I. Hydrogen atoms were omitted for clarity. Displacement ellipsoids are drawn at the 30% probability level.

**Fig. 2.**
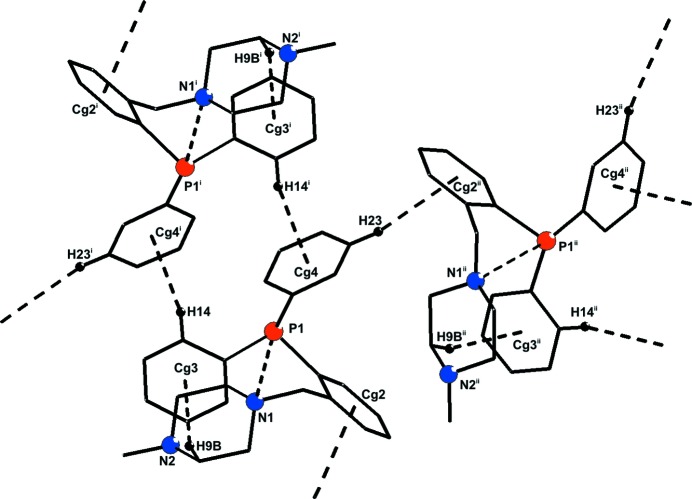
Intramolecular H···π and N···P interactions in the structure of I (shown as dashed lines). All the hydrogen atoms, except for those involved in hydrogen bonds, were omitted for clarity. Cg2, Cg3, and Cg4 are the centroids of the benzene rings C1—C6, C13—C18, and C19—C24, respectively. Symmetry codes: (i) -x + 2, -y + 1, -z; (ii) -x + 3/2, y - 1/2, -z + 1/2.

## supplementary crystallographic information

### Comment

We report here on the preparation and structural characterization in solution and the crystalline state of an asymetric triphosphane, PPh_2_[C_6_H_4_{CH_2_N(CH_2_CH_2_)_2_NMe}-2] (I).

Asymmetric organophosphorus(III) derivatives of the type PPh_2_*R*, where *R* is a substituent capable of intramolecular coordination, were reported in the literature (Pretorius *et al.*, 2004; Alberico *et al.*, 2007). Nevertheless, only in the symmetrical triorganophospane P[C_6_H_4_(CH_2_NMe_2_)-2]_3_ the nitrogen atom is coordinated to the phosphorous atom via N—P donor···acceptor interactions (Chuit *et al.*, 1993; Chandrasekaran *et al.*, 2002).

In the structure of (I) the nitrogen atom bonded to the benzyl fragment is coordinated to the phosphorous atom *trans* to a phenyl group. This induces chirality at the Ψ-trigonal-bipyramidal phosphorus centre, in addition to the planar chirality (the nitrogen atom acts as the pilot atom and the phenyl group as the chiral plane). (I) crystallizes in the racemic form. The (*R*_N_*A*_P_) isomer is shown in Fig. 1. The (*S*_N_*C*_P_) isomer is generated by symmetry with respect to the inversion centre.

The P—C bond lengths and C—P—C angles (Table 1) are in the range of those of triclinic PPh_3_ (Ziemer *et al.*, 2000). As expected, the P—C_Ph_ bond in *trans*-position to the P—N bond is slightly longer than the other P—C bond. The length of the P—N bond donor···acceptor interaction is in the range of values found in P[C_6_H_4_(CH_2_NMe_2_)-2]_3_ (Chandrasekaran *et al.*, 2002).

The structure displays intramolecular C–H···π bonds between one hydrogen atom of the piperazinyl group and a phenyl group (Fig. 2). Four intermolecular C–H···π bonds are established between the hydrogen atoms of the phenyl groups and π electrons of the benzene rings of neighbouring molecules. Geometrical parameters of the hydrogen bonds are listed in Table 2.

### Experimental

To a cooled solution of [2-{4-MeN(CH_2_CH_2_)_2_NCH_2_}C_6_H_4_]Li (2.55 g, 13 mmol) in tetrahydrofuran (thf) (203 K) was added dropwise a solution of PPh_2_Cl (2.40 ml, ρ = 1.23 g/ml, 13 mmol) in thf. The reaction mixture was stirred at 203 K for 2 h and was allowed to warm to room temperature. The solvent was removed under reduced pressure and over the resulting oily product was added dichloromethane. The obtained suspension was filtered and the dichloromethane was removed under reduced pressure. The remaining oily product solidified on addition of hexane. (I) was isolated as a colourless solid. Colourless crystals were obtained by the diffusion method from a dichloromethane/hexane mixture. Yield: 2.87 g (59%). mp 69 °C. ^1^H NMR (400 MHz, CDCl_3_): δ 1.96 (s, 3H, NMe), 2.26 (s,br, 8H, H_8,11_+H_9,10_), 3.62 (d, 2H, H_7_, ^4^J_PH_ = 0.8 Hz), 6.88 (m, 1H, H_6_), 7.09 (ddd, 1H, H_4_, ^3^J_HH_ = 7.5 Hz, ^4^J_HH_ = 1.3 Hz), 7.18 (m, 11H, P—C_6_H_5_ + H_5_), 7.29 (m, 1H, H_3_). ^13^C NMR (100 MHz, CDCl_3_): δ 45.86 (s, NMe), 52.15 (s, C_8,11_), 54.24 (s, C_9,10_), 61.95 (d, C_7_, ^3^J_PC_ = 15.5 Hz), 127.17 (s, C_4_), 128.14 (m, C_6_H_5_-*meta*+*para*), 128.32 (s, C_5_), 129.48 (d, C_3_, ^3^J_PC_ = 5.8 Hz), 133.55 (d, C_6_H_5_-*ortho*, ^2^J_PC_ = 19.6 Hz), 134.89 (s, C_6_), 137.03 (d, C_2_, ^2^J_PC_ = 15.9 Hz), 138.45 (d, C_6_H_5_-*ipso*, ^1^J_PC_ = 9.9 Hz), 143.76 (d, C_1_, ^1^J_PC_ = 24.2 Hz). ^31^P NMR (162 MHz, CDCl_3_): δ -16.1 (*s*).

### Refinement

Hydrogen atoms were placed in calculated positions with isotropic displacement parameters set at 1.2 times of those of the parent carbon atoms for aromatic and methylene hydrogen atoms, and at 1.5 times for hydrogen atoms of the methyl group.

### Crystal data

**Table:**

C_24_H_27_N_2_P	*F*(000) = 800
*M**_r_* = 374.45	*D*_x_ = 1.193 Mg m^−^^3^
Monoclinic, *P*2_1_/*n*	Melting point: 342 K
Hall symbol: -P 2yn	Mo *K*α radiation, λ = 0.71073 Å
*a* = 9.3689 (10) Å	Cell parameters from 3207 reflections
*b* = 14.6735 (16) Å	θ = 2.4–23.9°
*c* = 15.4362 (16) Å	µ = 0.14 mm^−^^1^
β = 100.849 (2)°	*T* = 297 K
*V* = 2084.2 (4) Å^3^	Block, colourless
*Z* = 4	0.30 × 0.26 × 0.21 mm

### Data collection

**Table:**

Bruker SMART APEX CCD area-detector diffractometer	3667 independent reflections
Radiation source: fine-focus sealed tube	2811 reflections with *I* > 2σ(*I*)
graphite	*R*_int_ = 0.064
φ and ω scans	θ_max_ = 25°, θ_min_ = 1.9°
Absorption correction: multi-scan (*SADABS*; Bruker, 2000)	*h* = −11→11
*T*_min_ = 0.959, *T*_max_ = 0.971	*k* = −17→17
14906 measured reflections	*l* = −18→18

### Refinement

**Table:**

Refinement on *F*^2^	Primary atom site location: structure-invariant direct methods
Least-squares matrix: full	Secondary atom site location: difference Fourier map
*R*[*F*^2^ > 2σ(*F*^2^)] = 0.085	Hydrogen site location: inferred from neighbouring sites
*wR*(*F*^2^) = 0.178	H-atom parameters constrained
*S* = 1.20	*w* = 1/[σ^2^(*F*_o_^2^) + (0.0598*P*)^2^ + 1.2183*P*] where *P* = (*F*_o_^2^ + 2*F*_c_^2^)/3
3667 reflections	(Δ/σ)_max_ = 0.001
245 parameters	Δρ_max_ = 0.25 e Å^−^^3^
0 restraints	Δρ_min_ = −0.25 e Å^−^^3^

### Special details

**Table:**

Geometry. All e.s.d.'s (except the e.s.d. in the dihedral angle between two l.s. planes) are estimated using the full covariance matrix. The cell e.s.d.'s are taken into account individually in the estimation of e.s.d.'s in distances, angles and torsion angles; correlations between e.s.d.'s in cell parameters are only used when they are defined by crystal symmetry. An approximate (isotropic) treatment of cell e.s.d.'s is used for estimating e.s.d.'s involving l.s. planes.
Refinement. Refinement of *F*^2^ against ALL reflections. The weighted *R*-factor *wR* and goodness of fit *S* are based on *F*^2^, conventional *R*-factors *R* are based on *F*, with *F* set to zero for negative *F*^2^. The threshold expression of *F*^2^ > σ(*F*^2^) is used only for calculating *R*-factors(gt) *etc*. and is not relevant to the choice of reflections for refinement. *R*-factors based on *F*^2^ are statistically about twice as large as those based on *F*, and *R*- factors based on ALL data will be even larger.

### Fractional atomic coordinates and isotropic or equivalent isotropic displacement parameters (Å^2^)

**Table:**

	*x*	*y*	*z*	*U*_iso_*/*U*_eq_	
C1	0.6682 (4)	0.5851 (2)	0.1668 (3)	0.0428 (9)	
C2	0.5236 (4)	0.6120 (2)	0.1377 (3)	0.0486 (10)	
C3	0.4311 (5)	0.6148 (3)	0.1980 (4)	0.0666 (13)	
H3	0.3349	0.6325	0.1794	0.08*	
C4	0.4792 (6)	0.5920 (3)	0.2845 (4)	0.0783 (16)	
H4	0.4153	0.5936	0.3239	0.094*	
C5	0.6207 (6)	0.5670 (3)	0.3133 (3)	0.0731 (14)	
H5	0.6535	0.5525	0.3724	0.088*	
C6	0.7139 (5)	0.5633 (3)	0.2549 (3)	0.0542 (11)	
H6	0.8099	0.5459	0.2749	0.065*	
C7	0.4705 (4)	0.6395 (3)	0.0433 (3)	0.0541 (11)	
H7A	0.4805	0.5884	0.0049	0.065*	
H7B	0.3683	0.6554	0.0348	0.065*	
C8	0.5243 (4)	0.7993 (2)	0.0655 (3)	0.0516 (10)	
H8A	0.5466	0.7889	0.1287	0.062*	
H8B	0.4223	0.8152	0.0494	0.062*	
C9	0.6161 (4)	0.8769 (2)	0.0421 (3)	0.0493 (10)	
H9A	0.5953	0.9316	0.0726	0.059*	
H9B	0.7181	0.8623	0.0615	0.059*	
C10	0.6156 (5)	0.8106 (3)	−0.0969 (3)	0.0603 (12)	
H10A	0.7175	0.7946	−0.0799	0.072*	
H10B	0.5951	0.8207	−0.1602	0.072*	
C11	0.5246 (5)	0.7335 (3)	−0.0750 (3)	0.0610 (12)	
H11A	0.4226	0.7479	−0.0948	0.073*	
H11B	0.5463	0.679	−0.1055	0.073*	
C12	0.6772 (5)	0.9677 (3)	−0.0734 (3)	0.0725 (14)	
H12A	0.6606	1.021	−0.0406	0.109*	
H12B	0.6525	0.9807	−0.1354	0.109*	
H12C	0.7777	0.9507	−0.0584	0.109*	
C13	0.8981 (4)	0.6775 (2)	0.1019 (2)	0.0374 (9)	
C14	0.9778 (4)	0.6993 (3)	0.0381 (3)	0.0526 (10)	
H14	0.9738	0.6612	−0.0105	0.063*	
C15	1.0624 (5)	0.7758 (3)	0.0448 (3)	0.0672 (13)	
H15	1.1167	0.7885	0.0016	0.081*	
C16	1.0676 (5)	0.8335 (3)	0.1143 (3)	0.0666 (13)	
H16	1.1241	0.886	0.1183	0.08*	
C17	0.9895 (5)	0.8137 (3)	0.1778 (3)	0.0626 (12)	
H17	0.9931	0.853	0.2255	0.075*	
C18	0.9055 (4)	0.7367 (3)	0.1725 (3)	0.0485 (10)	
H18	0.8532	0.7241	0.2167	0.058*	
C19	0.9139 (4)	0.4871 (2)	0.1368 (2)	0.0400 (9)	
C20	1.0627 (4)	0.4962 (3)	0.1523 (3)	0.0554 (11)	
H20	1.1033	0.5516	0.1405	0.066*	
C21	1.1529 (5)	0.4251 (3)	0.1850 (3)	0.0639 (12)	
H21	1.2532	0.4328	0.1942	0.077*	
C22	1.0960 (5)	0.3439 (3)	0.2037 (3)	0.0582 (11)	
H22	1.157	0.2964	0.2269	0.07*	
C23	0.9485 (5)	0.3325 (3)	0.1881 (3)	0.0546 (11)	
H23	0.9091	0.2769	0.2004	0.065*	
C24	0.8585 (4)	0.4026 (2)	0.1544 (2)	0.0461 (10)	
H24	0.7585	0.3935	0.143	0.055*	
N1	0.5526 (3)	0.7170 (2)	0.0195 (2)	0.0432 (8)	
N2	0.5876 (4)	0.8936 (2)	−0.0522 (2)	0.0534 (9)	
P1	0.78585 (10)	0.57472 (7)	0.08427 (6)	0.0391 (3)	

### Atomic displacement parameters (Å^2^)

**Table:**

	*U*^11^	*U*^22^	*U*^33^	*U*^12^	*U*^13^	*U*^23^
C1	0.044 (2)	0.032 (2)	0.054 (2)	−0.0038 (17)	0.0124 (18)	0.0048 (17)
C2	0.041 (2)	0.032 (2)	0.075 (3)	−0.0081 (17)	0.017 (2)	−0.0027 (19)
C3	0.053 (3)	0.046 (3)	0.109 (4)	−0.011 (2)	0.037 (3)	0.001 (3)
C4	0.101 (4)	0.046 (3)	0.109 (5)	−0.006 (3)	0.073 (4)	0.006 (3)
C5	0.109 (4)	0.048 (3)	0.072 (3)	0.010 (3)	0.043 (3)	0.013 (2)
C6	0.069 (3)	0.044 (2)	0.055 (3)	0.009 (2)	0.025 (2)	0.007 (2)
C7	0.037 (2)	0.044 (2)	0.075 (3)	−0.0056 (18)	−0.007 (2)	−0.005 (2)
C8	0.056 (3)	0.041 (2)	0.059 (3)	0.0023 (19)	0.014 (2)	−0.0035 (19)
C9	0.049 (2)	0.036 (2)	0.062 (3)	−0.0010 (18)	0.010 (2)	−0.0048 (19)
C10	0.071 (3)	0.064 (3)	0.045 (2)	−0.002 (2)	0.008 (2)	0.001 (2)
C11	0.065 (3)	0.056 (3)	0.054 (3)	0.001 (2)	−0.007 (2)	−0.009 (2)
C12	0.072 (3)	0.059 (3)	0.091 (4)	0.003 (2)	0.029 (3)	0.021 (3)
C13	0.034 (2)	0.037 (2)	0.039 (2)	0.0023 (16)	0.0011 (16)	0.0081 (17)
C14	0.050 (2)	0.054 (3)	0.054 (2)	−0.002 (2)	0.011 (2)	0.004 (2)
C15	0.059 (3)	0.070 (3)	0.076 (3)	−0.015 (2)	0.022 (3)	0.018 (3)
C16	0.055 (3)	0.056 (3)	0.082 (3)	−0.019 (2)	−0.003 (3)	0.012 (3)
C17	0.066 (3)	0.055 (3)	0.061 (3)	−0.018 (2)	−0.004 (2)	−0.006 (2)
C18	0.048 (2)	0.050 (2)	0.045 (2)	−0.0071 (19)	0.0030 (18)	0.0022 (19)
C19	0.043 (2)	0.041 (2)	0.035 (2)	−0.0044 (17)	0.0066 (16)	−0.0081 (16)
C20	0.047 (2)	0.049 (3)	0.068 (3)	−0.001 (2)	0.005 (2)	0.002 (2)
C21	0.042 (2)	0.061 (3)	0.084 (3)	0.006 (2)	0.001 (2)	0.003 (3)
C22	0.062 (3)	0.047 (3)	0.063 (3)	0.015 (2)	0.003 (2)	0.005 (2)
C23	0.073 (3)	0.040 (2)	0.053 (3)	0.001 (2)	0.018 (2)	0.0044 (19)
C24	0.045 (2)	0.040 (2)	0.054 (2)	−0.0047 (18)	0.0112 (19)	−0.0038 (18)
N1	0.0405 (18)	0.0381 (18)	0.0483 (19)	−0.0022 (14)	0.0012 (14)	−0.0042 (14)
N2	0.055 (2)	0.046 (2)	0.058 (2)	0.0053 (16)	0.0083 (17)	0.0103 (17)
P1	0.0366 (5)	0.0398 (6)	0.0404 (6)	−0.0027 (4)	0.0058 (4)	0.0008 (4)

### Geometric parameters (Å, °)

**Table:**

C1—C6	1.384 (5)	C12—N2	1.448 (5)
C1—C2	1.402 (5)	C12—H12A	0.96
C1—P1	1.841 (4)	C12—H12B	0.96
C2—C3	1.386 (6)	C12—H12C	0.96
C2—C7	1.504 (6)	C13—C14	1.381 (5)
C3—C4	1.368 (7)	C13—C18	1.386 (5)
C3—H3	0.93	C13—P1	1.829 (4)
C4—C5	1.367 (7)	C14—C15	1.366 (6)
C4—H4	0.93	C14—H14	0.93
C5—C6	1.369 (6)	C15—C16	1.360 (6)
C5—H5	0.93	C15—H15	0.93
C6—H6	0.93	C16—C17	1.361 (6)
C7—N1	1.458 (5)	C16—H16	0.93
C7—H7A	0.97	C17—C18	1.370 (5)
C7—H7B	0.97	C17—H17	0.93
C8—N1	1.452 (5)	C18—H18	0.93
C8—C9	1.510 (5)	C19—C20	1.376 (5)
C8—H8A	0.97	C19—C24	1.391 (5)
C8—H8B	0.97	C19—P1	1.839 (4)
C9—N2	1.450 (5)	C20—C21	1.376 (5)
C9—H9A	0.97	C20—H20	0.93
C9—H9B	0.97	C21—C22	1.359 (6)
C10—N2	1.449 (5)	C21—H21	0.93
C10—C11	1.493 (6)	C22—C23	1.368 (6)
C10—H10A	0.97	C22—H22	0.93
C10—H10B	0.97	C23—C24	1.368 (5)
C11—N1	1.453 (5)	C23—H23	0.93
C11—H11A	0.97	C24—H24	0.93
C11—H11B	0.97	N1—P1	3.051 (3)
			
C6—C1—C2	118.8 (4)	H12A—C12—H12B	109.5
C6—C1—P1	123.1 (3)	N2—C12—H12C	109.5
C2—C1—P1	118.0 (3)	H12A—C12—H12C	109.5
C3—C2—C1	118.8 (4)	H12B—C12—H12C	109.5
C3—C2—C7	120.6 (4)	C14—C13—C18	117.5 (4)
C1—C2—C7	120.6 (4)	C14—C13—P1	117.3 (3)
C4—C3—C2	121.0 (5)	C18—C13—P1	125.2 (3)
C4—C3—H3	119.5	C15—C14—C13	121.4 (4)
C2—C3—H3	119.5	C15—C14—H14	119.3
C5—C4—C3	120.3 (4)	C13—C14—H14	119.3
C5—C4—H4	119.9	C16—C15—C14	120.3 (4)
C3—C4—H4	119.9	C16—C15—H15	119.8
C4—C5—C6	119.7 (5)	C14—C15—H15	119.8
C4—C5—H5	120.1	C15—C16—C17	119.4 (4)
C6—C5—H5	120.1	C15—C16—H16	120.3
C5—C6—C1	121.4 (4)	C17—C16—H16	120.3
C5—C6—H6	119.3	C16—C17—C18	120.9 (4)
C1—C6—H6	119.3	C16—C17—H17	119.5
N1—C7—C2	111.1 (3)	C18—C17—H17	119.5
N1—C7—H7A	109.4	C17—C18—C13	120.5 (4)
C2—C7—H7A	109.4	C17—C18—H18	119.8
N1—C7—H7B	109.4	C13—C18—H18	119.8
C2—C7—H7B	109.4	C20—C19—C24	117.0 (4)
H7A—C7—H7B	108	C20—C19—P1	124.4 (3)
N1—C8—C9	110.2 (3)	C24—C19—P1	118.2 (3)
N1—C8—H8A	109.6	C19—C20—C21	121.6 (4)
C9—C8—H8A	109.6	C19—C20—H20	119.2
N1—C8—H8B	109.6	C21—C20—H20	119.2
C9—C8—H8B	109.6	C22—C21—C20	120.2 (4)
H8A—C8—H8B	108.1	C22—C21—H21	119.9
N2—C9—C8	111.3 (3)	C20—C21—H21	119.9
N2—C9—H9A	109.4	C21—C22—C23	119.6 (4)
C8—C9—H9A	109.4	C21—C22—H22	120.2
N2—C9—H9B	109.4	C23—C22—H22	120.2
C8—C9—H9B	109.4	C22—C23—C24	120.3 (4)
H9A—C9—H9B	108	C22—C23—H23	119.8
N2—C10—C11	111.5 (4)	C24—C23—H23	119.8
N2—C10—H10A	109.3	C23—C24—C19	121.2 (4)
C11—C10—H10A	109.3	C23—C24—H24	119.4
N2—C10—H10B	109.3	C19—C24—H24	119.4
C11—C10—H10B	109.3	C8—N1—C11	109.7 (3)
H10A—C10—H10B	108	C8—N1—C7	111.9 (3)
N1—C11—C10	110.6 (3)	C11—N1—C7	112.3 (3)
N1—C11—H11A	109.5	C12—N2—C10	111.1 (3)
C10—C11—H11A	109.5	C12—N2—C9	110.5 (3)
N1—C11—H11B	109.5	C10—N2—C9	108.7 (3)
C10—C11—H11B	109.5	C13—P1—C19	101.62 (16)
H11A—C11—H11B	108.1	C13—P1—C1	103.38 (16)
N2—C12—H12A	109.5	C19—P1—C1	100.26 (16)
N2—C12—H12B	109.5		
			
C6—C1—C2—C3	−0.8 (5)	C21—C22—C23—C24	−0.4 (6)
P1—C1—C2—C3	176.2 (3)	C22—C23—C24—C19	−1.2 (6)
C6—C1—C2—C7	177.8 (3)	C20—C19—C24—C23	1.9 (5)
P1—C1—C2—C7	−5.3 (5)	P1—C19—C24—C23	175.7 (3)
C1—C2—C3—C4	0.1 (6)	C9—C8—N1—C11	−57.1 (4)
C7—C2—C3—C4	−178.4 (4)	C9—C8—N1—C7	177.6 (3)
C2—C3—C4—C5	0.8 (7)	C10—C11—N1—C8	57.5 (4)
C3—C4—C5—C6	−1.1 (7)	C10—C11—N1—C7	−177.4 (3)
C4—C5—C6—C1	0.4 (7)	C2—C7—N1—C8	−67.7 (4)
C2—C1—C6—C5	0.5 (6)	C2—C7—N1—C11	168.4 (3)
P1—C1—C6—C5	−176.2 (3)	C11—C10—N2—C12	179.6 (3)
C3—C2—C7—N1	118.9 (4)	C11—C10—N2—C9	57.9 (4)
C1—C2—C7—N1	−59.6 (5)	C8—C9—N2—C12	−179.6 (3)
N1—C8—C9—N2	58.3 (4)	C8—C9—N2—C10	−57.6 (4)
N2—C10—C11—N1	−58.8 (5)	C14—C13—P1—C19	89.3 (3)
C18—C13—C14—C15	0.9 (6)	C18—C13—P1—C19	−93.1 (3)
P1—C13—C14—C15	178.7 (3)	C14—C13—P1—C1	−167.1 (3)
C13—C14—C15—C16	−1.3 (7)	C18—C13—P1—C1	10.5 (4)
C14—C15—C16—C17	1.0 (7)	C20—C19—P1—C13	−21.0 (4)
C15—C16—C17—C18	−0.2 (7)	C24—C19—P1—C13	165.6 (3)
C16—C17—C18—C13	−0.3 (6)	C20—C19—P1—C1	−127.1 (3)
C14—C13—C18—C17	−0.1 (6)	C24—C19—P1—C1	59.5 (3)
P1—C13—C18—C17	−177.7 (3)	C6—C1—P1—C13	−79.7 (3)
C24—C19—C20—C21	−0.9 (6)	C2—C1—P1—C13	103.5 (3)
P1—C19—C20—C21	−174.3 (3)	C6—C1—P1—C19	25.0 (3)
C19—C20—C21—C22	−0.7 (7)	C2—C1—P1—C19	−151.8 (3)
C20—C21—C22—C23	1.4 (7)		

### Hydrogen-bond geometry (Å, °)

**Table:**

*D*—H···*A*	*D*—H	H···*A*	*D*···*A*	*D*—H···*A*
C9—H9B···Cg3	0.97	2.91	3.835 (4)	160
C14—H14···Cg4^i^	0.97	2.74	3.651 (5)	166
C23—H23···Cg2^ii^	0.97	2.98	3.830 (5)	154

Symmetry codes: (i) −*x*+2, −*y*+1, −*z*; (ii) −*x*+3/2, *y*−1/2, −*z*+1/2.
